# 

**Published:** 1906-08-04

**Authors:** 


### BOOKS RECEIVED.

Baillière, Tindall, and Cox.

"Questions and Answers on Nursing." Fifth edition. By J. W. Martin, M.D.

"Applied Bacteriology." By C. G. Moor, M.A., and R. T. Hewlett, M.D. Third edition.

"Diseases of the Nose." By J. B. Ball, M.D. Fifth edition.

W. Green and Sons.

"Essentials of Human Physiology." By D. Noël Paton, M.D. Second edition.

Clinic Publishing Co., Chicago.

"Abbott's Alkaloïdal Digest."

Percy Lindley.

"Summer Holidays."

G. Rangecroft and Co., Clapham Junction.

"What is Truth?" By A Woman.

Simpkin, Marshall and Co.

"The Rise and Progress of Hydropathy in England and Scotland." By Richard Metcalfe.

Percival Jones, Limited, Birmingham.

"City of Birmingham, Report of the Housing Committee."

H. Kimpton.

"The Science and Art of Prescribing." By E. H. Colbeck, B.A., and Arnold Chaplin, B.A. Second edition.

J. B. Lippincott Co.

"A Nurse's Handbook of Medicine." By J. Norman Henry, M.D.

Walter Scott Publishing Co., Limited.

"Guide to Household Nursing." By Miss S. A. Andrew.

### Medical Vacancies.

Bracebridge Asylum, Lincolnshire.—Senior Assistant Medical Officer.

Brighton, Sussex County Hospital.—House Physician.

Brighton Throat and Ear Hospital, Church Street, Queen's Road.—Non-resident House Surgeon.

Colchester, Essex and Colchester General Hospital.—House Physician.

Egyptian Government, Ministry of Education.—Professor of Midwifery and Gynæcology. Also Medical Tutor and Registrar to Kasr-el-Ainy Hospital.

Evelina Hospital for Sick Children, Southwark Bridge Road, S.E.—Physician to Out-patients.

Mount Vernon Hospital for Consumption and Diseases of the Chest, Hampstead and Northwood.—Resident Medical Officer.

North-Eastern Hospital for Children, Hackney Road, Bethnal Green, E.—Medical Officer in charge of Electrical Department.

Sherburn (Ancient) Hospital, near Durham.—Medical Officer.

Sunderland Infirmary.—Two House Surgeons.

Weston-super-Mare Hospital.—House Surgeon.

Wisbech, North Cambridgeshire Hospital.—Resident Medical Officer.

Wolverhampton and Staffordshire General Hospital.—Assistant House Surgeon.

### NOTICE TO CORRESPONDENTS.

All MSS., Letters, Books for Review, and other matters intended for the Editor should be addressed The Editor, "The Hospital," The Hospital Buildings, 28 & 29 Southampton Street, Strand, W.C.

The Editor cannot undertake to return rejected MS., even when accompanied by stamped directed envelope.

**Notice to Advertisers and Subscribers.**

All Advertisements, Orders for Copies of The Hospital, and Business Communications should be addressed to **THE MANAGER (Not to the Editor)**, The Hospital, 28 & 29 Southampton Street, Strand London, W.C.

The Cost of Subscription, post free, is as follows:—Per week, 3½d.; per quarter, 3s. 3d.; per half-year, 6s. 6d.; per annum, 13s. Foreign and Colonial:—Per annum, 19s. 6d., payable, in all cases, in advance.

**NOTICE.—Vol. XXXIX. of "The Hospital" (October 1905 to March 1906), in handsome cloth binding, will shortly be on sale at the office of this Journal, price 10s. 6d. Cases for binding the Numbers contained in this Volume 2s. Index to Vol. XXXIX., 3½d., post free.**

**The Monthly Part for August is now ready. Price 1s., by post, 1s. 3d.**

### Publications.

### NEURASTHENIA, Clinical Lectures on.

By THOMAS D. SAVILL, M.D. Lond. 2nd Edition.

*Physician to the Hospital for Diseases of the Nervous System, &c. &c.*

"To every thoughtful practitioner it will be immensely useful."—JOURN. OF BAL. & CLIM. "Goes very fully into the details of treatment."—THE LANCET.

H. J. GLAISHER, 57 WIGMORE STREET, W. **5s. net; 5s. 4d. by post.**

NOW READY.—ALL BOOKSELLERS.
Crown 8vo. 320 Pages. Cloth, Price 3s. 6d. Twenty-one Illustrations.

**THE RISE AND PROGRESS OF HYDROPATHY IN ENGLAND AND SCOTLAND.** By RICHARD METCALFE, The Hydro, Richmond, Surrey, Author of "Essays and Notes on Hydro-therapeutics," "Life of Vincent Priessnitz," &c.
London: SIMPKIN, MARSHALL & Co.

### IMPORTANT TO HOSPITAL SECRETARIES.

**JUST PUBLISHED. Price 8s. 6d. net.**

### Surgical Dressings Stock Book.

This type of book is used by some of the leading Institutions in the country, and has been found to be of the greatest value as a ready means of noting the details of Surgical Dressings, &c., and comparing the items from day to day or month to month. By its use extravagance or waste in any ward is easily detected.

*PUBLISHED BY*

**THE SCIENTIFIC PRESS, LTD.,**
28 & 29 Southampton Street, Strand, London, W.C.

### CLEFT PALATE AND HARE-LIP:

**The Earlier Operation on the Palate.**

By **EDMUND OWEN,** M.B., F.R.C.S., Consulting Surgeon St. Mary's Hospital, and to the Hospital for Sick Children, Great Ormond Street, London.
Pp. 111, with 32 Illustrations. Just Published. Price **2s. 6d.** net.
"A most valuable contribution to the literature of this subject."—*British Medical Journal.*
London: **BAILLIÈRE, TINDALL, & COX, 8 Henrietta Street, W.C.**

### WORKS BY DAVID WALSH, M.D.

**RÖNTGEN RAYS IN MEDICAL WORK.** *Third Edition.* 12s. 6d.

**THE HAIR AND ITS DISEASES.** 2s. 6d. net. A Standard Monograph. Baillère, Tindall, & Cox, London.

**GOLDEN RULES OF SKIN PRACTICE.** 1s. net. *Second Edition.* Wright & Co., Bristol.

**AGE AND OLD AGE: Handbook.** 2s. 6d. R. A. Everett & Co., London.

JUST PUBLISHED. Price **2s. 6d.** net.

### CARCINOMA OF THE RECTUM.

By F. SWINFORD EDWARDS, F.R.C.S.,
Surgeon to the West London Hospital; Senior Surgeon to St. Peter's Hospital for Stone, and to St. Mark's Hospital for Fistula, &c.

London: BAILLIÈRE, TINDALL, & COX, 8 Henrietta Street, Covent Garden.

THE MOST EFFECTUAL BANDAGE FOR THE CURE OF ULCERS AND OTHER DISEASES OF THE LEG.

Now ready. 10s. 6d. net; 10s. 11d. post free.

### THE CONSUMPTIVE WORKING MAN:

### What can Sanatoria do for Him?

BY

**NOEL DEAN BARDSWELL,** M.D., M.R.C.P., F.R.S. (Edin.),

Medical Superintendent, King Edward VII. Sanatorium.

Of all Booksellers, or of THE SCIENTIFIC PRESS, LTD., 28/29 Southampton Street, Strand, London, W.C.

### HOSPITAL ACCOUNTS.

### THE UNIFORM SYSTEM OF ACCOUNTS

**For Hospitals and all Classes of Public Institutions.**

With Special Forms of Account and Specimen Rulings of a Complete Set of Books.

**By Sir HENRY BURDETT, K.C.B.**

New Edition. Royal 8vo. cloth, 4s. net; 4s. 4d. post free.

### ACCOUNT BOOKS FOR HOSPITALS & PUBLIC INSTITUTIONS

**Designed in Accordance with the Uniform System of Accounts for Hospitals, &c.**

A Descriptive List of Books and Prices post free on application.

THE SCIENTIFIC PRESS, LTD.,
28 & 29 SOUTHAMPTON STREET,
STRAND, LONDON, W.C.

